# Predictors of return to work after shoulder injuries: The influence of health perception and job characteristics

**DOI:** 10.1371/journal.pgph.0007563

**Published:** 2026-11-06

**Authors:** Sahand Nik Asa, Elham Mirzamohammadi, Morteza Nakhaei Amroodi, Pouria Tabrizian, Saber Mohammadi, Zahra Asadgol, Negin Kassiri

**Affiliations:** 1 Occupational Medicine Research Center, Iran University of Medical Sciences, Tehran, Iran; 2 Department of Occupational Medicine, School of Medicine, Iran University of Medical Sciences, Tehran, Iran; 3 Bone and Joint Reconstruction Research Center, Shafa Orthopedics Hospital, Iran University of Medical Sciences, Tehran, Iran; 4 Department of Orthopedics, School of Medicine, Iran University of Medical Sciences, Tehran, Iran; Universiti Malaya, MALAYSIA

## Abstract

Shoulder injuries are prevalent among working populations and may significantly delay return to work (RTW). Identifying predictors of successful RTW is crucial for designing effective interventions. This study aimed to assess the RTW status and its determinants among patients with shoulder injuries. In this retrospective cohort study with prospective follow-up, 340 patients with shoulder injuries— such as acromioclavicular joint dislocation, clavicle fractures, and shoulder instability—treated between 2016 and 2020 were included. Data were obtained from medical records and structured telephone interviews. Demographic and clinical variables, health-related quality of life (SF-12), and perceived support from employer and colleagues were assessed. Multivariate logistic regression was used to identify predictors of RTW. Among 340 participants, 67.9% returned to their previous job, 14.1% returned with modifications, 14.4% obtained new employment, and 3.5% did not return to work. Multivariate analysis showed that coworker support (OR=1.88, p < 0.001) was positively associated with RTW, whereas lower employer support (OR=0.585, p < 0.001), lower SF‑12 scores (OR=0.895, p = 0.023), and white‑collar occupation (OR=0.015, p < 0.001) were associated with reduced likelihood of RTW. In addition, health insurance coverage and certain injury types were significantly related to RTW outcomes. RTW after shoulder injuries is mainly influenced by multiple occupational, clinical, and psychosocial factors. Workplace support, overall health status, and job characteristics play important roles in facilitating occupational reintegration. Strengthening rehabilitation programs and workplace support systems may improve RTW outcomes among injured workers.

## 1. Introduction

Shoulder pain is among the most prevalent musculoskeletal complaints in the general population, with a point prevalence ranging between 18% to 26% and an annual incidence estimated at approximately 0.9% to 2.5% [1–3]. It is the third most common musculoskeletal symptom leading to medical consultations in primary care settings, surpassed only by back and knee pain [4]. The clinical presentation of shoulder disorders is often multifactorial, involving tendinopathies, rotator cuff tears, impingement syndrome, instability, and fractures, all of which can substantially impair functional ability and quality of life [5,6]. Shoulder injuries often lead to persistent and debilitating symptoms that interfere with daily functioning at home and work, while simultaneously contributing to substantial socioeconomic costs through prolonged work disability, and reduced productivity [7–9]. Shoulder pain and limited mobility negatively impact not only physical functioning but also sleep quality and psychological health, especially among working-age individuals, and these impairments are frequently accompanied by increased healthcare utilization, reduced occupational performance, prolonged absenteeism, and in some cases, premature workforce exit due to an inability to return to work (RTW) [4,10,11].

Return to work after shoulder injury is influenced by a range of factors beyond physical recovery, including rehabilitation protocols, psychosocial conditions, job demands, and workplace relationships. In some cases, non-physical factors such as motivation, job satisfaction, and perceived benefits may have a stronger impact on RTW decisions than physical limitations [12]. In a cohort study by Feltri et al., nearly 73% of patients returned to work after arthroscopic rotator cuff repair, although individuals engaged in physically intensive occupations often experience longer delays in returning to work, greater reductions in level of employment, and higher rates of job loss, all of which can hinder their ability to resume a satisfactory pre-injury lifestyle [13]. Similarly, Ting et al. found that individuals who remained employed between the time of injury and surgery of rotator cuff showed the highest likelihood of returning to work in any capacity. Those with lower physical demands in their pre-injury occupations were more likely to resume their previous work roles [14]. Pichené-Houard et al. reported that return-to-work patterns were categorized as stable, unstable, or absent, with nearly 68% of participants holding physically demanding jobs. Key predictors of these trajectories included preoperative self-rated health, presence of a repaired rotator cuff tear, and the physical intensity of the job [15]. Additionally, a clinical trial by Anderson et al. demonstrated that Integrating a digital exercise program into conventional rehabilitation for workers’ compensation patients undergoing rotator cuff repair led to faster return to work, improved pain outcomes, enhanced functional recovery, and fewer complications [16].These findings highlight the complex and multifactorial nature of RTW after shoulder injuries, underscoring the importance of job type, rehabilitation modality, and psychosocial context.

Given the impact of delayed return to work on both individual well-being and societal productivity, assessing the timing of RTW and identifying its key determinants is essential for overcoming barriers and facilitating a timely reintegration into the workforce. Although the clinical characteristics of shoulder injuries are generally similar across populations, return-to-work outcomes may be influenced by contextual factors such as labor market conditions, workplace policies, and job characteristics. Despite the clinical and occupational significance of this issue, there is a lack of comprehensive studies exploring RTW outcomes and associated factors among individuals with shoulder injuries in Iran. In light of this gap, the present study aims to evaluate RTW status and its influencing factors in patients recovering from common shoulder injuries, including acromioclavicular joint dislocation, clavicle fractures, and shoulder instability.

## 2. Materials and methods

### 2.1. Ethics statement

All procedures performed in this study were conducted in accordance with the ethical principles outlined in the Declaration of Helsinki. The study protocol was reviewed and approved by the Medical Research Ethics Committee of Iran University of Medical Sciences (Approval Code: IR.IUMS.FMD.REC.1400.149).

Eligible participants were approached during their routine follow-up visits at the orthopedic clinic after their initial treatment. The study objectives and procedures were explained to each participant, and written informed consent was obtained before enrollment. Participants provided consent for access to relevant information in their medical records and for subsequent follow-up contact. No additional consent was obtained for subsequent follow-up assessments because these assessments were part of the procedures described in the original consent.

The authors had access to identifiable patient information only for recruitment and follow-up purposes. All study data were de-identified prior to analysis, and personal identifiers were not included in the analytical dataset. Professional confidentiality and data privacy were maintained throughout the study. Participation was entirely voluntary, and participants could decline to answer any follow-up question or discontinue participation without any consequences for their medical care. Telephone follow-up was conducted only after obtaining permission to contact participants, and the purpose of each call was explained before the assessment. No financial compensation or study-related costs were involved.

### 2.2. Study design and data collection

This retrospective cohort study with prospective follow-up was conducted at a tertiary care hospital in Tehran. All patients who had been treated for eligible shoulder injuries at the study center between 01/03/2016 and 31/03/2020 were initially identified through a review of medical records. A total of 482 patients were identified during this period. The predefined inclusion and exclusion criteria were then applied, resulting in 340 eligible patients who constituted the final study cohort. No random sampling or selection based on a target sample size was performed; all patients who met the eligibility criteria were included in the study.

The study population consisted of patients who received either surgical or non-surgical treatment for shoulder injuries, including acromioclavicular joint dislocation, clavicle fracture, or shoulder instability. Patients were eligible if they were 18–65 years of age, had been employed at the time of injury, had no history of previous shoulder surgery, and were available for follow-up. Patients with medical conditions considered likely to substantially affect return-to-work outcomes were excluded.

Patients were approached during their routine follow-up visits at the orthopedic clinic after their initial treatment. At this visit, the study objectives and procedures were explained, and written informed consent was obtained before any study-specific data collection. Consent included permission to access relevant medical-record information and to contact the participant for subsequent follow-up. Thus, informed consent was obtained once at enrollment; no additional consent was required for subsequent follow-up contacts.

Baseline demographic and clinical information was obtained from the medical records, including age, sex, occupation, marital status, education level, and type of shoulder injury. Information that was not available in the medical records, including relevant work-related characteristics and return-to-work information, was obtained directly from the participants during follow-up.

Return-to-work status was assessed prospectively beginning approximately two months after the injury and was subsequently reassessed at approximately monthly intervals for up to one year after the injury, or until the participant returned to work, whichever occurred first. The two-month starting point was selected because an initial period of approximately two months of medical leave is commonly used for musculoskeletal injuries in the Iranian healthcare and insurance context, making this a clinically relevant time point for the initial assessment of occupational reintegration.

Follow-up assessments were conducted during routine orthopedic clinic visits whenever participants attended the clinic. When a participant did not attend a scheduled or routine follow-up visit, the same assessment was conducted by telephone. This approach allowed RTW status to be monitored approximately monthly while minimizing loss to follow-up. Follow-up was discontinued once the participant returned to work; participants who had not returned to work continued to be followed until one year after the injury.

During follow-up, participants were asked about their return-to-work status and related factors, including duration of work absence, reason for work discontinuation, time to the first full-time workday, type of insurance, perceived insurance support, employer support, coworker support, and job satisfaction. Duration of work absence was defined as the total number of workdays lost because of the injury. Time to the first full-time workday was defined as the number of days from hospital discharge or completion of the initial treatment to the first day on which the participant resumed full-time work. Job titles before the injury and after return to work were assessed and classified by an occupational medicine specialist. Occupations were also classified according to their physical work demands.

Of the 340 participants, 32 received non-surgical treatment, while the remaining participants underwent surgical treatment according to their clinical indications. The study did not determine or modify the treatment approach; treatment was provided by the treating orthopedic team as part of routine clinical care.

In Iran, workers covered by social or occupational insurance schemes may receive wage replacement benefits or financial support during periods of work absence related to injury. Therefore, information on insurance type and perceived insurance support was collected as potential determinants of return-to-work outcomes.

### 2.3. Health-related quality of life assessment

To assess quality of life, participants completed the Persian version of the 12-Item Short Form Health Survey (SF-12), a widely validated self-report instrument developed by Ware, Kosinski, and Keller in 1996 [17]. The SF-12 evaluates eight health domains: general health, physical functioning, and role limitations due to physical or emotional problems, bodily pain, vitality, social functioning, and mental health. It generates two summary scores—physical and mental component summaries—ranging from 0 to 100, with higher scores indicating better health status.

The psychometric validity and reliability of the SF-12 have been confirmed in several populations. Test–retest reliability coefficients are reported as 0.89 for the physical and 0.76 for the mental domains. Construct and empirical validity have been demonstrated by Ware et al. and Kontodimopoulos et al. In Iran, Montazeri et al. conducted a validation study of the Persian version of the SF-12 [18], confirming its cultural adaptability and psychometric soundness. Test-retest reliability yielded coefficients of 0.73 and 0.72 for the physical and mental components, respectively. Convergent validity analysis showed strong correlations between subscale scores and their respective overall domain scores, supporting the scale’s validity in Iranian populations.

### 2.4. Statistical analysis

Data analysis was performed using SPSS software version 24. Descriptive statistics, including frequency, percentage, mean, and standard deviation, were used to summarize demographic and clinical variables. To assess the normality of continuous data, the Kolmogorov–Smirnov test was applied. Depending on the distribution of the data, parametric tests (such as t-test and one-way ANOVA) or their non-parametric equivalents (such as Mann–Whitney U and Kruskal–Wallis tests) were used to examine group differences. The primary outcome of the study was return-to-work (RTW) status. During follow-up, participants were categorized as: (1) returned to their previous job, (2) returned to work with job modifications, (3) returned to a new job, or (4) did not return to work. For regression analysis, RTW status was dichotomized into RTW (categories 1–3) and non-RTW (category 4). For bivariate analysis, comparisons were made between participants who returned to work and those who did not, across all independent variables. Variables that demonstrated a statistically significant association (p < 0.05) in univariate analyses were subsequently entered into a multivariate logistic regression model to identify independent predictors of return to work. Statistical significance was set at p < 0.05 for all tests.

## 3. Results

A total of 340 patients with shoulder injuries were included in the study. The majority of participants were male (85.9%), while females accounted for 14.1%. The mean age of patients was 34.8 years (range: 19–59). In terms of marital status, 52.5% were married, 44.1% were single, and 3.5% were categorized as other (e.g., divorced). Regarding smoking habits, 62.1% reported no tobacco use, whereas 37.9% were current smokers. With respect to education, 18.2% had less than a high school diploma, 25.0% completed high school, 10.3% held an associate degree, 42.1% had a bachelor’s degree, and only 4.4% had postgraduate qualifications (master’s or doctorate).

Occupational and clinical characteristics showed that 55.6% of patients were employed in white-collar jobs, while 44.4% worked in blue-collar occupations. In terms of insurance coverage, 76.8% of participants were insured, predominantly by social security, while 23.2% had no insurance. Overall, nearly half of the patients had at least one underlying medical condition, which may have influenced their recovery and return-to-work outcomes. Most patients (90.6%) received surgical treatment, and only 9.4% were managed non-surgically. Additionally, 47.4% of patients reported at least one comorbid condition, while 52.6% had no comorbidities. Rehabilitation interventions were received by 70.3% of participants, whereas 29.7% did not undergo any form of rehabilitation. Table 1 summarizes the demographic and clinical characteristics of the study population. The de-identified dataset supporting the findings of this study is provided in S1 Data.

**Table 1:** Demographic and clinical characteristics of study participants (n = 340).

Variable	Category	Frequency (n)	Percentage (%)
**Gender**	Male	292	85.9
	Female	48	14.1
**Marital Status**	Married	178	52.5
	Single	150	44.1
	Other (e.g., divorced)	12	3.5
**Smoking Status**	Non-smoker	211	62.1
	Smoker	129	37.9
**Education Level**	Less than high school	62	18.2
	High school diploma	85	25.0
	Associate degree	35	10.3
	Bachelor’s degree	143	42.1
	Master’s degree	11	3.2
	Doctorate degree	4	1.2
Job Category*	Blue-collar	151	44.4
	White-collar	189	55.6
**Insurance**	Insured	261	76.8
	Uninsured	79	23.2
**Underlying disease**	With underlying disease	161	47.4
	No underlying disease	179	52.6
**Treatment Type**	Surgical	308	90.6
	Non-surgical	32	9.4
**Rehabilitation Received**	Yes	239	70.3
	No	101	29.7

* ***Blue-collar*** *jobs* refer to occupations involving manual labor or physically demanding tasks, whereas ***white-collar*** *jobs* include non-manual or office-based roles typically requiring higher educational qualifications [19].

As illustrated in Fig 1, the distribution of shoulder injuries among participants showed that shoulder instability was by far the most common type, accounting for 73% of cases, followed by clavicle fracture (21%) and acromioclavicular joint dislocation (6%). Regarding treatment modalities, surgical interventions were predominant, with Bankart repair representing the majority at 59.4% of cases. Other surgical techniques included Open Reduction and Internal Fixation)ORIF((20.6%), Weaver-Dunn (3.2%), Hook plate fixation (2.9%), Latarjet (2.6%), and Close reduction (2.4%). Conservative (non-surgical) management accounted for 8.8% of patients. These findings indicate that instability-related injuries were the most prevalent and were primarily managed through Bankart procedures.

**Fig 1:**
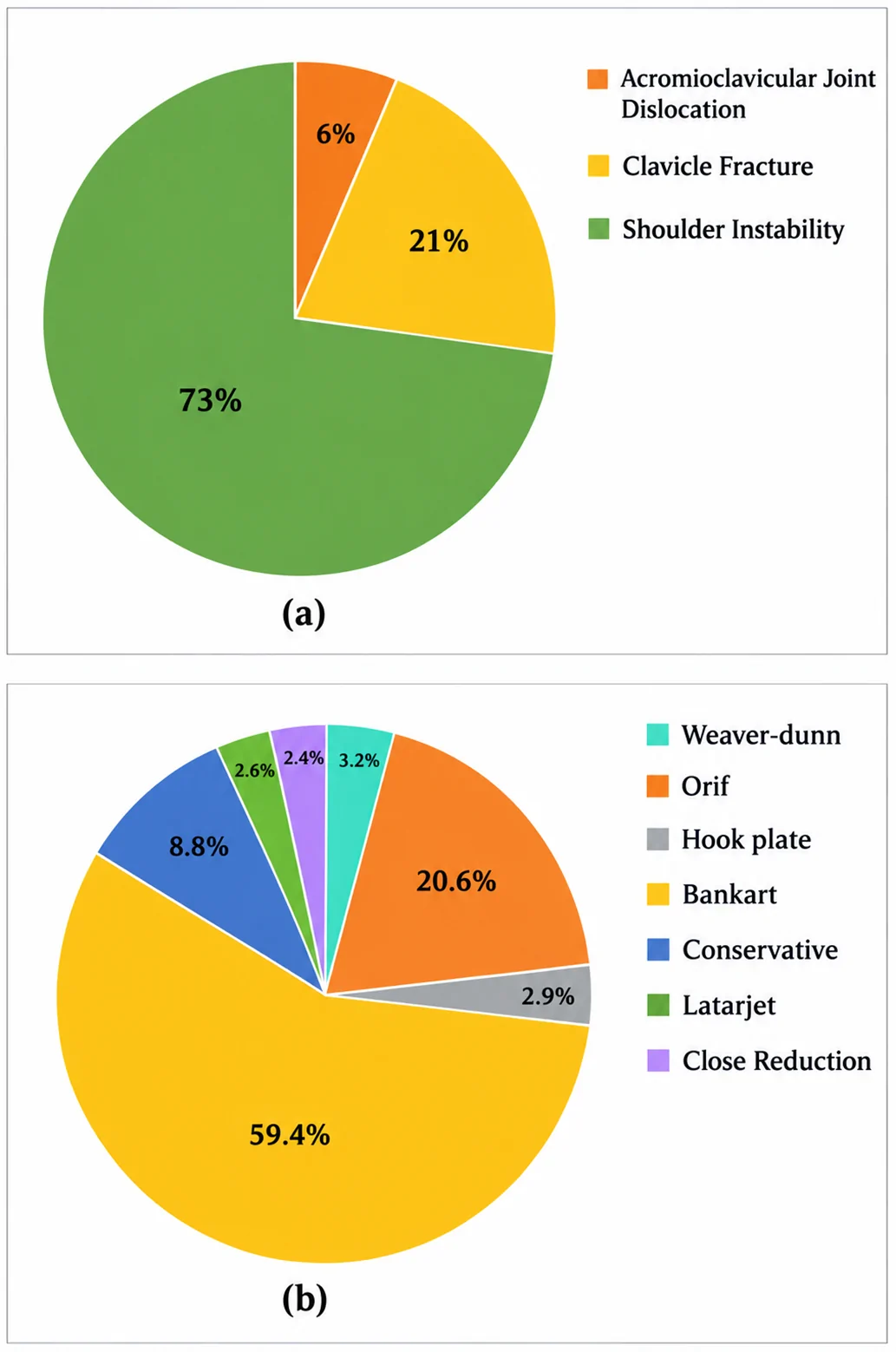
Percentage of (a) Shoulder Injuries and (b) Therapeutic and Surgical Procedures among participants.

According Table 2, the average work experience was 10.64 ± 9.61 years, and the mean weekly working hours were 55.16 ± 13.56 hours. Regarding post-injury work-related variables, the mean duration until the first full-time workday was 69.64 ± 46.11 days, while the overall duration of work absence averaged 54.94 ± 38.01 days. Participants reported moderate levels of insurance support (mean = 4.45 ± 3.06), and relatively high levels of employer and coworker support, with mean scores of 7.81 ± 2.79 and 7.68 ± 2.57, respectively. Job satisfaction scores were moderate, with a mean of 6.71 ± 2.51. Clinical and rehabilitation-related variables indicated a mean hospital stay of 2.77 ± 1.39 days. The duration of rehabilitation averaged 2.84 ± 9.96 days, with a mean of 19.70 ± 9.07 rehabilitation sessions per patient. Health-related quality of life, measured by the SF-12 total score, had a mean value of 34.43 ± 6.76, reflecting a moderate overall health status among participants.

**Table 2:** Descriptive statistics of demographic, occupational, clinical, and health-related variables.

Variable	Minimum	Maximum	Mean	Standard Deviation (SD)
**Age (years)**	19.00	59.00	34.80	10.18
**Height (cm)**	152.00	198.00	176.39	8.66
**Weight (kg)**	52.00	110.00	77.85	11.84
**Work Experience (years)**	0.00	38.00	10.64	9.61
**Weekly Working Hours**	5.00	90.00	55.16	13.56
**First Full-Time Workday Post-Injury (days)**	1.00	270.00	69.64	46.11
**Duration of Work Absence (days)**	3.00	180.00	54.94	38.01
**Insurance Support (score 0–10)**	0.00	10.00	4.45	3.06
**Employer Support (score 0–10)**	0.00	10.00	7.81	2.79
**Coworker Support (score 0–10)**	1.00	10.00	7.68	2.57
**Length of Hospital Stay (days)**	0.00	8.00	2.77	1.39
**Number of Rehabilitation Sessions**	10.00	40.00	19.70	9.07
**SF-12 Total Score**	18.00	47.00	34.43	6.76

Table 3 demonstrates the return-to-work (RTW) status according to demographic and clinical characteristics. Among the 340 participants, the majority returned to work following their shoulder injuries: 231 patients (68%) resumed their previous jobs, 48 (14%) returned to a modified position, 49 (14%) started a new job, and 12 patients (4%) did not return to work at all. Age appeared to influence RTW outcomes, with younger participants (<40 years) demonstrating a higher likelihood of returning to their original roles, whereas individuals over 50 years showed increased rates of non-return. Gender differences were also observed, as male participants were more likely to resume their previous employment, while female participants exhibited higher proportions of modified work or non-RTW. The p-values further highlight which demographic and clinical variables were significantly associated with RTW status. Specifically, age group (p = 0.001), gender (p = 0.001), job category (p = 0.001), type of shoulder injury (p = 0.03), procedure type (p = 0.04), underlying disease (p = 0.001), insurance type (p = 0.02), and quality of life (p = 0.04) demonstrated significant associations with RTW outcomes. In contrast, marital status, education level, and rehabilitation measures were not significantly related (p > 0.05). These findings suggest that both clinical and occupational characteristics play a critical role in predicting return-to-work trajectories among patients with shoulder injuries.

**Table 3:** Return-to-work status by demographic and clinical characteristics.

		Returned to Same Job (%)	Returned to Modified Job (%)	Returned to New Job (%)	Did Not Return to Work (%)	p-value
**Age Group**	< 20 years	14 (6.0)	0 (0.0)	2 (4.1)	0 (0.0)	0.001
	21–30 years	90 (38.9)	17 (35.4)	9 (18.4)	2 (16.7)	
	31–40 years	88 (38.1)	13 (27.1)	20 (40.8)	0 (0.0)	
	41–50 years	29 (12.5)	13 (27.1)	10 (16.3)	0 (0.0)	
	> 50 years	10 (14.3)	5 (10.4)	8 (20.4)	10 (83.3)	
**Gender**	Male	198 (85.7)	46 (95.8)	44 (89.8)	4 (33.3)	0.001
	Female	33 (14.3)	2 (4.2)	5 (10.2)	8 (66.7)	
**Marital Status**	Married	118 (51.1)	25 (52.1)	29 (59.2)	7 (58.3)	0.48
	Single	108 (46.7)	23 (47.9)	16 (32.7)	2 (16.7)	
	Other1	5 (2.1)	0 (0.0)	4 (8.1)	3 (25.0)	
**Education Level**	Below High School	18 (7.8)	14 (29.2)	23 (46.9)	7 (58.3)	0.21
	High School Diploma	59 (25.5)	9 (18.7)	12 (24.5)	5 (41.7)	
	Associate Degree	29 (12.5)	4 (8.3)	2 (4.1)	0 (0.0)	
	Bachelor’s Degree	112 (48.5)	21 (43.8)	10 (20.4)	0 (0.0)	
	Master’s Degree	9 (3.9)	0 (0.0)	2 (4.1)	0 (0.0)	
	Doctoral Degree	4 (1.7)	0 (0.0)	0 (0.0)	0 (0.0)	
**Job Category**	White-Collar	58 (25.1)	42 (87.5)	42 (85.7)	9 (75.0)	0.001
	Blue-Collar	173 (74.9)	6 (12.5)	7 (14.3)	3 (25.0)	
**Type of Shoulder Injury**	Acromioclavicular Joint Dislocation	12 (5.2)	2 (4.2)	6 (12.2)	0 (0.0)	0.03
	Clavicle Fracture	40 (17.3)	12 (25.0)	16 (32.7)	4 (33.3)	
	Shoulder Instability	179 (77.5)	34 (70.8)	27 (55.1)	8 (66.7)	
**Procedure Type**	Weaver-Dunn	9 (3.9)	0 (0.0)	2 (4.1)	0 (0.0)	0.04
	ORIF	40 (17.3)	10 (20.8)	16 (32.6)	4 (33.3)	
	Hook Plate	6 (2.6)	2 (4.2)	2 (4.1)	0 (0.0)	
	Bankart Repair	141 (61.0)	28 (58.3)	25 (51.0)	8 (66.7)	
	Conservative Treatment	20 (8.6)	8 (16.7)	2 (4.1)	0 (0.0)	
	Latarjet Procedure	7 (3.0)	0 (0.0)	2 (4.1)	0 (0.0)	
	Closed Reduction	8 (3.5)	0 (0.0)	0 (0.0)	0 (0.0)	
**Rehabilitation measures**	No	71 (30.7)	18 (37.5)	10 (20.4)	2 (16.7)	0.21
	Yes	160 (69.3)	30 (62.5)	39 (79.6)	10 (83.3)	
**Underlying disease**	With underlying disease	113 (48.9)	14 (29.2)	24 (49.0)	10 (83.4)	0.001
	No underlying disease	118 (51.1)	34 (70.8)	25 (51.0)	2 (16.6)	
**Insurance type**	Social Security	111 (48.0)	30 (62.5)	16 (32.6)	8 (66.7)	0.02
	Health	63 (27.2)	6 (12.5)	10 (20.4)	0 (0.0)	
	Armed Forces	11 (4.8)	0 (0.0)	6 (12.2)	0 (0.0)	
	Uninsured	46 (20.0)	12 (25.0)	17 (34.7)	4 (33.3)	
**Quality of life**	Weak	12 (5.2)	6 (12.5)	15 (30.6)	5 (41.7)	0.04
	Average	93 (40.3)	24 (50.0)	32 (65.3)	7 (58.3)	
	Good	126 (54.5)	18 (37.5)	2 (4.1)	0 (0.0)	
**Total**		**231 (67.9)**	**48 (14.1)**	**49 (14.4)**	**12 (3.5)**	

Demographic characteristics, including marital status and education level, also played a notable role in RTW patterns. Married participants and those with higher educational attainment, particularly individuals holding a bachelor’s degree or higher, were more likely to return to their original jobs or transition to a new role, suggesting that social support and professional qualifications may facilitate occupational recovery. In contrast, patients with lower education levels tended to assume modified duties or, in some cases, did not return to work. Job category further influenced outcomes; white-collar employees more frequently shifted to modified or new positions, whereas blue-collar workers predominantly returned to their previous roles, likely reflecting differences in job flexibility and physical demands.

Clinical and treatment-related factors significantly affected RTW outcomes. Surgical procedures such as Bankart repair and ORIF were associated with lower rates of returning to work; similarly, patients with underlying diseases also showed lower return-to-work rates. Participation in rehabilitation programs positively correlated with RTW, highlighting the importance of structured post-injury recovery. Importantly, patients who returned to their original jobs reported higher health-related quality of life (HRQoL) scores on the SF-12 survey, whereas those who transitioned to modified or new positions or did not return to work exhibited lower scores. These findings suggest that resuming one’s previous employment may contribute directly to improved perceived quality of life, potentially through the restoration of routine, social engagement, financial stability, and personal fulfillment.

Multivariate logistic regression analysis was conducted to identify independent predictors of return to work among patients with shoulder injuries (Table 4). Several workplaces, clinical, and health-related variables demonstrated significant associations with the study outcome. Employer support (OR = 0.585, 95% CI: 0.449–0.762, p < 0.001), coworker support (OR = 1.881, 95% CI: 1.343–2.634, p < 0.001), and SF-12 score (OR = 0.895, 95% CI: 0.813–0.985, p = 0.023) were significantly associated with the outcome variable. In addition, the presence of underlying disease significantly affected outcomes, particularly diabetes and chronic kidney disease. Patients with acromioclavicular joint dislocation had significantly different outcomes compared with the reference injury category (OR = 31.135, 95% CI: 2.353–411.980, p = 0.009). Longer hospital stay (OR = 1.424, 95% CI: 1.008–2.012, p = 0.045) and health insurance coverage (OR = 13.683, 95% CI: 2.155–86.874, p = 0.006) were also significantly associated with the outcome. White-collar occupation remained a significant predictor (OR = 0.015, 95% CI: 0.003–0.071, p < 0.001).

**Table 4:** Results of logistic regression analysis including coefficients, standard errors, significance levels, and odds ratios for each predictor variable entered into the model.

Variables	B	S.E.	Wald	df	p-value	Odds Ratio	95% CI for OR
Age	0.036	0.063	0.319	1	0.572	1.036	0.916 – 1.172
Gender (Male)	1.041	1.006	1.072	1	0.301	2.833	0.395 – 20.341
Work Experience	-0.005	0.060	0.007	1	0.935	0.995	0.885 – 1.119
Insurance Support	0.077	0.129	0.351	1	0.553	1.080	0.838 – 1.391
Employer Support	-0.536	0.135	15.804	1	<0.001	0.585	0.449 - 0.762
Coworker Support	0.632	0.172	13.518	1	<0.001	1.881	1.343 – 2.634
Quality of life (SF-12)	-0.111	0.049	5.133	1	0.023	0.895	0.813 – 0.985
Primary Occupation (White-Collar)	-4.172	0.776	28.888	1	<0.001	0.015	0.003 – 0.071
**Underlying Disease**			12.718	4	0.013		
Diabetes	-5.523	1.697	10.598	1	.001	.004	0.000-0.111
Hypertension	-3.087	1.633	3.575	1	.059	.046	0.002-1.120
Chronic kidney disease	-3.591	1.446	6.170	1	.013	.028	0.002-0.469
Liver disorders	-2.686	1.381	3.785	1	.052	.068	0.005-1.020
**Shoulder Injury**			10.302	2	0.006		
Acromioclavicular Joint Dislocation	3.438	1.318	6.809	1	0.009	31.135	2.353 – 411.980
Clavicle Fracture	1.453	1.128	1.660	1	0.198	4.278	0.469 – 39.043
Length of Hospital Stay	0.354	0.176	4.015	1	0.045	1.424	1.008 – 2.012
Time to First Full-Time Workday	0.013	0.010	1.831	1	0.176	1.013	0.994 – 1.033
Duration of Work Absence	-0.012	0.012	0.957	1	0.328	0.988	0.965 – 1.012
**Type of Insurance**			11.745	3	0.008		
Social Security Insurance	-0.603	0.600	1.009	1	0.315	0.547	0.169 – 1.774
Health Insurance	2.616	0.943	7.696	1	0.006	13.683	2.155 – 86.874
Armed Forces Insurance	1.674	1.050	2.541	1	0.111	5.334	0.681 – 41.786
Constant	5.463	3.653	2.236	1	0.135	235.723	

Overall, these findings highlight that RTW is a multifactorial outcome, shaped by social, occupational, clinical, and insurance-related factors. The results emphasize the importance of comprehensive rehabilitation and workplace support, as well as tailored interventions for patients with comorbidities or in physically or cognitively demanding roles, to facilitate successful occupational reintegration.

## 4. Discussion

This cohort study, conducted among 340 patients with shoulder injuries, including acromioclavicular joint dislocation, clavicle fractures, and shoulder instability, and did not include patients undergoing shoulder arthroplasty, provides new insights into the multifactorial determinants of return to work (RTW). The majority of patients successfully resumed employment, although variations were observed depending on demographic, occupational, and clinical characteristics. Notably, the highest RTW rates were observed among patients undergoing shoulder arthroscopy, consistent with prior evidence suggesting that arthroscopic procedures—due to their minimally invasive nature, reduced postoperative pain, and faster functional recovery—facilitate earlier and higher occupational reintegration compared to open surgeries or shoulder arthroplasty. Retrospective data from arthroscopic rotator cuff repair (ARCR) indicated nearly universal RTW at a median of eight weeks postoperatively, although RTW was delayed in patients with physically demanding jobs or those involved in Workers’ Compensation systems [20]. Systematic review and meta-analysis results further support this, demonstrating RTW rates of approximately 83.8% at a mean of 6.6 months post-ARCR [21]. Similarly, Latarjet procedures (either arthroscopic or open) have demonstrated high RTW rates exceeding 89%, with return occurring between roughly 8.7 and 34.8 weeks post-surgery [22]. By contrast, shoulder arthroplasty studies report RTW rates ranging from 56% to 65% for reverse total shoulder arthroplasty and 71% to 93% for total shoulder arthroplasty, with return times averaging 2–4 months depending on occupational demands [23]. In less invasive interventions such as subacromial decompression, diagnostic arthroscopy, and exercise therapy, RTW rates have exceeded 80% at 24 months [24]. Collectively, these findings—aligned with our cohort results—underscore that timely and effective clinical and rehabilitative management, tailored to injury type, surgical procedure, and patient characteristics, plays a central role in facilitating occupational reintegration following shoulder injury.

Younger individuals (<40 years) exhibited higher rates of return to work (RTW) following shoulder injuries, whereas RTW rates declined noticeably in those over 50 years of age. This pattern is consistent with prior studies indicating that rehabilitation and functional recovery are generally slower in older adults due to reduced physical capacity and the presence of comorbidities [25,26]. Additionally, men demonstrated the highest rates of return to their previous jobs, whereas women experienced higher proportions of modified work or non-return to work. This differences may reflect cultural factors, family responsibilities, physical capacity as well as gender roles influencing occupational choices and recovery outcomes [27]. Interestingly, despite these observed trends in descriptive analyses, the logistic regression results of the present study indicated that age, male gender, work experience, and insurance support were not statistically significant predictors of RTW. This aligns with prior evidence, including a systematic review by Hoving et al. (2010), which found that demographic and work-related factors such as age and work experience were not consistently associated with RTW outcomes [28]. These findings suggest that while age and gender may influence overall trends in occupational reintegration, they may not independently determine RTW when accounting for other social, occupational, and clinical factors. Furthermore, based on the results of the logistic regression analysis, workplace-related factors demonstrated differential impacts on return-to-work (RTW) outcomes among patients with shoulder injuries. Interestingly, neither work experience nor insurance support emerged as significant predictors of RTW, suggesting that the mere duration of prior employment or perceived financial coverage may not independently facilitate occupational reintegration. This aligns with previous research indicating that demographic and work-related factors, such as tenure or insurance, do not consistently predict RTW after musculoskeletal injuries. For instance, a study by Collie et al. (2019) found that while insurance claims data provide insights into injury-related absences, they do not reliably predict individual RTW outcomes [29]. Although prior studies often failed to establish a consistent link between insurance and RTW, our findings suggest that the type of insurance may be a more relevant determinant than insurance status alone. Specifically, individuals covered by health insurance had significantly higher odds of returning to work (OR=13.68, 95% CI: 2.16–86.87, p = 0.006), whereas social security and armed forces insurance were not associated with RTW. This suggests that the influence of insurance is not uniform but may depend on the scope of benefits and support provided by specific insurance schemes. In Iran, workers covered by social insurance or occupational injury insurance schemes may be eligible for wage-replacement benefits and financial support during periods of work absence following injury. Such compensation mechanisms may influence return-to-work decisions by reducing the immediate economic pressure to resume work. Although the present study assessed insurance type and perceived insurance support, detailed information regarding compensation amounts, duration of benefits, and eligibility criteria was not available. Therefore, the potential impact of wage-replacement policies on RTW outcomes could not be fully evaluated and warrants further investigation in future studies. Accordingly, healthcare providers and policymakers should consider not only whether individuals are insured but also the type of insurance coverage when designing RTW interventions [30].

In contrast, social support within the workplace played a pivotal role. Higher coworker support significantly increased the odds of RTW (OR = 1.881, 95% CI: 1.343–2.634, p < 0.001), similarly, higher employer support was associated with increased odds of RTW (OR = 1.585, 95% CI: 0.449–1.762, p < 0.001), highlighting the importance of peer encouragement, assistance, and the social environment in promoting occupational reintegration. Health-related quality of life (HRQoL), as measured by the SF-12 total score, emerged as a significant predictor of return to work (RTW) in our cohort. The logistic regression analysis indicated that higher SF-12 scores were associated with increased likelihood of RTW (OR = 1.895, 95% CI: 0.813–1.985, p = 0.023), suggesting that patients with better overall physical and mental health are more capable of resuming occupational duties following shoulder injuries. This finding aligns with prior studies [31,32] demonstrating that HRQoL is a strong determinant of post-injury occupational reintegration, as individuals with higher physical function and lower pain perception are more likely to achieve functional recovery and maintain work participation. Also, this underscores the relevance of patients’ self-perceived health in facilitating occupational reintegration.

Our study identified that underlying diseases significantly reduce the likelihood of returning to work (RTW) following shoulder injuries. Previous research has shown that diabetic patients exhibited a fourfold increased risk of developing adhesive capsulitis, a common shoulder disorder, compared to non-diabetic individuals [33,34]. In addition, other studies have reported that CKD has been associated with negative outcomes after shoulder arthroplasty, including higher rates of infection and transfusion [35]. Further research with larger cohorts and more precise characterization of comorbidities is needed to better understand their impact on RTW outcomes. These findings underscore the importance of considering clinical comorbidities in the rehabilitation and management of patients with shoulder injuries. Tailored interventions that address these underlying conditions may improve functional recovery and facilitate RTW [36]. Unexpectedly, white-collar workers in our cohort had a lower likelihood of RTW (OR = 0.015; 95% CI: 0.003–0.071; p < 0.001). This contrasts with previous literature [37] and may be attributed to higher task-specific demands on shoulder mobility, older age distribution, or psychosocial factors unique to the study population. The nature of white-collar roles—typically involving prolonged sitting, cognitive complexity, and limited physical flexibility—may complicate early return despite clinical recovery. Moreover, structural demands or job responsibilities common in white-collar settings might delay RTW compared to more adaptable blue-collar roles. While direct literature on shoulder injury RTW by job type is limited, broader occupational health research acknowledges differing physical demands and psychosocial dynamics between white- and blue-collar jobs, which can influence recovery trajectories and disability risk [38,39]. However, a systematic review that examined return-to-work outcomes in individuals with hand injuries found no significant difference between white-collar and blue-collar occupations in returning to work [40]. Although, this discrepancy highlights the need for condition-specific studies, as findings from hand injury populations may not directly generalize to shoulder injuries.

### 4.1. Limitations and strengths

Despite the novel insights provided by this cohort study, several limitations should be acknowledged. First, the single-center design and geographically limited sample may restrict the generalizability of the findings to broader populations. Second, the relatively short follow-up period of approximately two months post-treatment may have precluded the capture of delayed return-to-work (RTW) or long-term changes in occupational status. Third, while efforts were made to mitigate potential recall bias by conducting follow-up assessments relatively soon after treatment and cross-referencing key data with medical records, a significant portion of the data, particularly regarding RTW status and psychosocial variables, was collected via telephone interviews. This reliance on self-report introduces the potential for recall bias, which could affect the accuracy of reported outcomes. Forth, although some psychosocial factors were collected, others such as occupational stress and detailed mental health status were not systematically evaluated throughout the study, potentially influencing RTW outcomes. Additionally, variability in injury severity, surgical procedures, and rehabilitation protocols may have contributed to heterogeneity in results. Furthermore, detailed information regarding rehabilitation interventions, including the type of physical therapy, home exercise programs, timing of rehabilitation initiation, treatment intensity, and patient adherence, was not available. As these factors may influence functional recovery and return-to-work outcomes, future studies should evaluate them in greater detail. A significant limitation of this study is the absence of objective measures to quantify initial injury severity. As severity can strongly influence RTW outcomes and act as a confounder, its omission from our analysis prevents us from fully isolating the effects of the predictors under investigation. Moreover, the small number of participants within certain subgroups (e.g., specific comorbidities or insurance types) may have limited statistical power for subgroup analyses. Future research should address these limitations by conducting multicenter studies with larger and more diverse populations, incorporating long-term follow-up, and systematically assessing psychosocial, occupational, and clinical factors. Furthermore, stratified analyses based on job type, insurance coverage, and rehabilitation protocols could provide more targeted recommendations. Cross-cultural and international comparative studies may also enhance understanding of how contextual factors, including labor regulations and social support systems, influence RTW outcomes. Addressing these gaps will help refine predictive models and guide the development of tailored interventions to optimize occupational reintegration following shoulder injuries.

## 5. Conclusion

This cohort study highlights key factors influencing return to work (RTW) following shoulder injuries. While most patients resumed employment, outcomes were shaped by a combination of clinical, demographic, and occupational factors. Notably, minimally invasive procedures, better health-related quality of life, and supportive workplace environments were associated with higher RTW, whereas underlying diseases, as well as employment in white-collar roles, reduced RTW likelihood. In summary, this study reinforces that successful RTW after shoulder injury is a multifactorial process requiring coordinated medical, rehabilitative, and workplace strategies. Targeted interventions addressing both clinical and occupational determinants can enhance functional recovery, shorten work absence, and improve long-term workforce participation, ultimately supporting patient well-being and socioeconomic productivity.

## Supporting information

S1 Data De-identified study dataset in SPSS format. (SAV)
